# Supplementary material for: Linear programming-based stabilization and synchronization of positive complex networks with dynamic link subsystems
Source: PLoS One. 2025 May 15;20(5):e0321582. doi: 10.1371/journal.pone.0321582 (PMC12080841; doi:10.1371/journal.pone.0321582)
Supplement: Attachment — Submitted filename: Plos one review.pdf [file pone.0321582.s001.pdf]

This paper provides a new perspective on the field by constructing a positive complex network model that includes dynamically linked subsystems. An innovative coupling term is designed and a related controller is proposed to achieve stability and synchronization of the network. Through the application of linear programming and copositive Lyapunov functions, this paper provides a feasible design, analysis, and calculation method, which is relatively new in the study of positive complex networks. Although the contributions of this paper are significant, authors are advised to consider the following improvements before final acceptance for publication:

1. The author should proofread the manuscript thoroughly to ensure that all mathematical symbols and formulas have been correctly presented.
2. In the preliminaries, the description of the set $\mathfrak{S}=\{1,2,\ldots,J\}$, $J\in\mathcal{N}^{+}$. The symbol $\mathcal{N}^{+}$ gives no indication, please check it.
3. There are two different design forms of coupling terms involved in this paper. Please further explain the differences between the two design forms.
4. The arguments about the positivity of the system in this paper all start from a specific moment t_0. I suggest the author providing a more detailed explanation of the positive proof starting from time t_0.
5. What are the fundamental differences between the synchronization problem of positive complex networks discussed in this paper and that of traditional complex dynamic networks?
