# Supplementary material for: Linear programming-based stabilization and synchronization of positive complex networks with dynamic link subsystems
Source: PLoS One. 2025 May 15;20(5):e0321582. doi: 10.1371/journal.pone.0321582 (PMC12080841; doi:10.1371/journal.pone.0321582)
Supplement: Attachment — Submitted filename: elsarticle-template-num.pdf [file pone.0321582.s002.pdf]

Response to Reviewers

No.: PONE-D-25-00197
Title: Linear programming-based stabilization and synchronization of positive complex networks with dynamic link subsystem
Authors: Shouting Hong, Junfeng Zhang, Gang Zheng, Haoyue Yang, Aslam Bhatti Uzair

Dear Editor,

We would like to express our great appreciation to the editor and reviewers for their valuable and very helpful comments and suggestions. We have carefully considered all the comments raised by the Reviewers and revised the paper accordingly. In addition, we have made some other corrections to improve the quality and readability of the paper. All changes have been highlighted in blue font. We now submit a new revised version to you. We hope that the revision meets the expectation of reviewers, and is suitable for publication in Plos One. If you have any requirements about the paper, please do not hesitate to contact us.
Best regards,
Yours sincerely,
The authors.

In the following, we provide detailed explanations of all the changes made in the present revised version.

To Academic Editor

We would like to thank the Academic Editor for spending his/her time to assess the paper. Our responses to the Academic Editor's comments are as follows:

Comments: *Two reviews have been received for the submission, and both reviewers express a positive assessment of the work. However, they also provide several suggestions for improving its quality. These suggestions include: 1) clarifying the key differences between the synchronization problem discussed in this paper and those in traditional complex dynamic networks; 2) providing a more detailed explanation of the positive proof; and 3) providing a thorough description of the simulation results. The authors are encouraged to address these points.*
Response: Thank you for giving us this opportunity to revise our paper. All comments have been carefully addressed and the corresponding corrections have been made.

To Reviewer #1

We would like to thank the reviewer for spending his/her time to assess the paper. Our responses to the reviewer's comments are as follows:

Comment 1: *In the introduction, individual places can be further refined to improve the reading experience of the paper.*

Response: Thanks for your helpful comments. We have optimized the logical structure in the introduction to ensure the coherence and logic of the discussion.

"This includes not only the development of new theories and methods, but also the exploration of synchronization strategies that can effectively deal with positive constraints."

"To effectively address the modeling and control of complex nonlinear systems, T-S fuzzy systems have emerged as a significant tool. T-S fuzzy systems characterize the behavior of the systems using a set of "IF-THEN" fuzzy rules, each corresponding to a linear subsystem [14]. This approach breaks down complex nonlinear issues into multiple local linear problems, thus the modeling and control processes are simplified. Therefore, the combination of synchronization framework and fuzzy systems can more effectively analyze and solve synchronization problems in CNs."

"Despite these remarkable advances, the synchronization of fuzzy positive MJCNs (PMJCNs) remains challenging."

Comment 2: *Some missing symbols in the manuscript. Please check carefully.*

Response: We would like to thank the reviewer for reading this paper carefully. Some symbols have been added in the revised version.

Comment 3: *In Theorem 1, "hold for each i, j" is inconsistent with "hold for each p, q" in Theorem 2 and Theorem 3. Please check and correct.*

Response: Thank you for your attention to detail. We have examined it carefully and made changes in the revised version.

Comment 4: *The equation (35) provides a design for a controller. Does it have any impact on synchronization or on Theorem 3? Some statements could be made about it.*

Response: Equation (35) does play an important role in controller design. This is essential for achieving synchronization within the system. Based on your comments, we have made corresponding modifications in Theorem 3 to reflect the influence of this key factor.

"**Theorem 3** If there exist constants $\underline{\alpha} > 0$, $\overline{\alpha} > 0$, $\kappa > 0$, $\mu_0 > 0$, $\mu_1 > 0$, $\mu_2 > 0$, $\mu_3 > 1$, $\gamma > 0$, $\mathbb{R}^n$ vectors $z_{np}^{(\iota)} \succ 0$, $\tilde{z}_{np}^{(\iota)} \succ 0$, $\eta_p \succ 0$, $\eta_q \succ 0$, and $\mathbb{R}^N$ vectors $\xi_p \succ 0$, $\xi_q \succ 0$ such that the conditions (10), (22c), (22e), (22f), and

$$\widetilde{A}_{bp} + \varepsilon \widetilde{F}_{bp} + \widetilde{B}_{bp} \sum_{\iota=1}^{n} \mathbf{1}_n^{(\iota)} \tilde{z}_{np}^{(\iota)\top} + \mu_0 I_n \succeq 0 \tag{1a}$$

$$A_{bp} - \widetilde{A}_{bp} - \widetilde{B}_{bp} \sum_{\iota=1}^{n} \mathbf{1}_n^{(\iota)} \tilde{z}_{np}^{(\iota)\top} + \varepsilon (F_{bp} - \widetilde{F}_{bp}) + \mu_1 I_n \succeq 0, \tag{1b}$$

$$A_{bp} + B_{bp} \sum_{\iota=1}^{n} \mathbf{1}_n^{(\iota)} z_{np}^{(\iota)\top} + \varepsilon F_{bp} + \mu_2 I_n \succeq 0, \tag{1c}$$

$$\underline{\delta} \leq \mathbf{1}_n^{(\iota)\top} \widetilde{B}_{bp}^{\top} \eta_p \leq \overline{\delta}, \tag{1d}$$

$$A_{bp}^{\top} \eta_p + \underline{\alpha} \sum_{\iota=1}^{n} z_{np}^{(\iota)} + \epsilon F_{bp}^{\top} \eta_p + \sum_{q=1}^{J} \lambda_{pq} \eta_q + \mu_3 C_{bp}^{\top} \mathbf{1}_n \prec 0, \tag{1e}$$

$$(A_{bp}^{\top} - \widetilde{A}_{bp}^{\top} - \overline{\delta}\sum_{\iota=1}^{l}\widetilde{z}_{np}^{(\iota)} + \epsilon(F_{bp}^{\top} - \widetilde{F}_{bp}^{\top}))\eta_p + \mu_3(C_{bp} - \widetilde{C}_{bp})^{\top}\mathbf{1}_n \prec 0, \tag{1f}$$

hold for each $p, q \in \mathfrak{S}$, then the system (5) is positive and reaches the synchronization with L_1-gain performance under the coupling term (21) and controllers (33) and (35) with

$$\widetilde{K}_{np} = \sum_{\iota=1}^{n}\mathbf{1}_n^{(\iota)}\widetilde{z}_{np}^{(\iota)\top}, K_{np} = \sum_{\iota=1}^{n}\mathbf{1}_n^{(\iota)}z_{np}^{(\iota)\top}."$$

Comment 5: *It would be beneficial if the author could offer a more detailed description of the simulation results.*

Response: We have described the simulation results in more detail.

"Figs. 4 and 5 show the simulations of the state trajectories $x_i(t)$ and $L_i(t), i = 1, 2, 3, 4, 5, 6$, respectively. It can be seen from Figs. 4 and 5 that the state trajectories of nodes and links are non-negative respectively and converge asymptotically to zero. The smoothness of convergence indicates that the controller has good performance in terms of stability and response time."

"Fig. 6 shows the simulations of the state trajectories $x_i(t)$ and $s(t), i = 1, 2, 3, 4, 5, 6$, Fig. 7 is the states of LSs, and Fig. 8 shows the tracking error of the six following nodes with respect to the leader $s(t)$ under the action of the controller (35). It can be observed from Fig. 8 that the tracking error of each node gradually approaches zero, which indicates that the synchronization controller has achieved the synchronization performance."

To Reviewer #2

We would like to thank the reviewer for spending his/her time to assess the paper. Our responses to the reviewer's comments are as follows:

Comment 1: *The author should proofread the manuscript thoroughly to ensure that all mathematical symbols and formulas have been correctly presented.*
Response: *Thank you. We have re-proofread the whole text carefully, especially all the mathematical symbols and formulas in the revised version.*

Comment 2: *In the preliminaries, the description of the set is not clear, please check it.*
Response: *Thank you for your reading our paper carefully. We have revisited and clarified the definition of all sets in the revised version.*

Comment 3: *There are two different design forms of coupling terms involved in this paper. Please further explain the differences between the two design forms.*
Response: *This is an important issue. Remark 5 is modified in the revised version to illustrate the problem.*
*"***Remark 5** *When analyzing the dynamic behavior of fuzzy PMJCNs, it is crucial to consider the dynamic characteristics of both NSs and LSs. This increases the complexity of the stability analysis. In Theorem 2, the coupling relationship is embodied in the correlation term involving the link state in NSs and the coupling term involving the node state in LSs. However, the coupling term design proposed in Theorem 1 is mainly concerned with the stability of LSs and the coupling term is independent of the node state. This design form allows us to handle link dynamics independently without considering the impact of the node state. This coupling item design is suitable for those cases that the link dynamics has a significant impact on the overall system performance, but the correlation with the node state is not strong. In contrast, the coupling term design in Theorem 2 is more general because it takes into account the effect of node states on the coupling term."*

Comment 4: *The arguments about the positivity of the system in this paper all start from a specific moment. I suggest the author providing a more detailed explanation of the positive proof starting from time.*
Response: *Thank you for your comments, we have provided Remark 6 in the revised version.*
*"***Remark 6** *In this paper, the positivity of the system is taken for any initial time. Since the state equation of NSs contains the state variable of LSs, it is not feasible to prove that the state of the system is non-negative at any time directly by checking the coefficient matrix of the systems. Therefore, an indirect but rigorous method is adopted in this paper. First, it is proved that from any initial time, the coefficient matrix of the differential equation of LSs satisfies the positive system condition, thus ensuring that the state vector at the next time is non-negative. We can then argue recursively that for all non-negative link initial states, the state of LSs will remain non-negative. Thus, it is derived that the state of NSs will also remain non-negative for any given non-negative initial state. This method not only ensures the rigorism of the demonstration process, but also provides an effective analytical framework for verifying the positivity of complex coupled systems."*

Comment 5: *What are the fundamental differences between the synchronization problem of positive complex networks discussed in this paper and that of traditional complex dynamic networks?*

Response: *Thank you for your comments, we have provided Remark 8 in the revised version.*
"**Remark 8** *This paper focuses on the synchronization issue of PCNs, where the connectivity and the network topology evolve dynamically over time. This characteristic is reflected through the proposed LSs model. The synchronization design of traditional CNs typically employs static topological graphs to represent fixed interconnections among nodes and concentrates on the synchronization of nodal dynamic behaviors. This paper needs to consider both node dynamics and link dynamics. Therefore, the corresponding synchronization problem is more challenging. Furthermore, a core feature of positive complex networks is that their state variables remain non-negative under all non-negative initial conditions. Such a positivity constraint is generally not considered in traditional CNs."*
